# P-823. Navigating Staphylococcus aureus Bloodstream Infections in Pediatrics: A Comprehensive Analysis

**DOI:** 10.1093/ofid/ofae631.1015

**Published:** 2025-01-29

**Authors:** Santhanam Naguthevar, Vibhor Tak, Kumar S Abhishek, Daisy Khera

**Affiliations:** AIIMS JODHPUR, Jodhpur, Rajasthan, India; AIIMS Jodhpur, Jodhpur, Rajasthan, India; AIIMS, Jodhpur, Rajasthan, India; AIIMS, Jodhpur, Rajasthan, India

## Abstract

**Background:**

*Staphylococcus aureus* bacteraemia poses substantial morbidity and mortality risks in children, with infection rates ranging from 1.5 to 3.5 per 1000 hospitalizations. Diagnosis and management of Staph. aureus is challenging. This study aims to delineate the spectrum of Staph. aureus bloodstream infections (BSI) in paediatric patients, aiding in the development of more effective management strategies.

**Methods:**

Conducted at AIIMS, Jodhpur, India, from February 2022 to January 2024, this prospective observational study gathered clinical and microbiological data from 82 patients with Staphylococcus aureus BSI.

**Results:**

In this study, the most commonly affected age group was children under 5 years, constituting 39.02% of cases, followed by those aged 11 to 15 years (31.71%), 16 to 20 years (17.07%), and 6 to 10 years (12.19%).

In the observed clinical syndromes, the most common was bone and joint infections, affecting 34.15% of patients, followed by healthcare-associated pneumonia (HAP) at 30.49%. Meningitis was observed in 13.41% of cases, while community-acquired pneumonia (CAP) accounted for 17.07%. Central line-associated bloodstream infections (CLABSI) were reported in 7.32% of patients, with skin and soft tissue infections (SSTI) noted in 3.66% of cases. Urinary tract infections (UTI) represented the least common, affecting 1.22% of patients.

Methicillin Resistant Staphylococcus aureus was present in 54.88% of cases, followed by Methicillin Sensitive Staphylococcus aureus at 40.24%, and Vancomycin Resistant Staphylococcus aureus at 4.88%.

In terms of outcomes, the majority of patients, constituting 75.61%, experienced improvement in their condition. Unfortunately, 21.95% of patients succumbed to the infection, while a small proportion, accounting for 2.44%, were lost to follow-up.

**Conclusion:**

MRSA underscores the challenges posed by antimicrobial resistance. Our comprehensive analysis of *Staphylococcus aureus* bloodstream infection reveals multifaceted challenges in its management..

**Disclosures:**

**All Authors**: No reported disclosures

